# Implementation of Microwave Circuits Using Stereolithography

**DOI:** 10.3390/polym14081612

**Published:** 2022-04-15

**Authors:** Germán Torregrosa-Penalva, Héctor García-Martínez, Ángela E. Ortega-Argüello, Alberto Rodríguez-Martínez, Arnau Busqué-Nadal, Ernesto Ávila-Navarro

**Affiliations:** 1 Communication Engineering Department, Miguel Hernández University of Elche, 03202 Elche, Spain; angela.ortega02@alu.umh.es (Á.E.O.-A.); arodriguez@umh.es (A.R.-M.); arnau.busque@alu.umh.es (A.B.-N.); 2 Materials Science, Optical and Electronic Technology Department, Miguel Hernández University of Elche, 03202 Elche, Spain; mhector@umh.es (H.G.-M.); eavila@umh.es (E.Á.-N.)

**Keywords:** 3D-printing, additive manufacturing, dielectric permittivity, microwave circuits, stereolithography, ultrasonic characterization

## Abstract

In this work, the use of additive manufacturing techniques through stereolithography for the manufacture of high-frequency circuits and devices is presented. Both the resin and the 3D printer used in this research are general-purpose commercial materials, not specifically intended for the implementation of microwave networks. The manufacturing and metallization procedures used to produce substrates for the design of planar microwave circuits are described, introducing the characterization process carried out to determine the electrical properties of the resin used. The ultrasonic techniques that allow the structural analysis of the manufactured substrates are also described. The electrical characterization provides a relative dielectric permittivity of 3.25 and a loss tangent of 0.03 for the resin used. In addition, the structural analysis shows a homogeneity and a finish of the manufactured parts that is not achievable using fused deposition modeling techniques. Finally, as a proof of concept, the design and manufacture of a complex geometry stepped impedance filter on a multi-height substrate using stereolithography techniques is presented, which allows for reducing the size of the traditional implementation of the same filter while maintaining its high-frequency response performance.

## 1. Introduction

In recent years, 3D printing or additive manufacturing (AM) technology has become popular in different sectors. AM allows the rapid design and manufacture of 3D objects from a computer aided design (CAD) file [1,2], without the need for using specific tools to implement each prototype [3]. Its wide success is also due to its low cost, easy handling and the possibility of creating complex structures, which are not feasible to implement using traditional machining techniques. Therefore, this technology has aroused great interest in the academic world and in a wide variety of technological industries, such as aerospace, medicine or electronics [4,5,6,7,8], opening new lines of scientific research and creating innovative prototypes with a very low manufacturing cost. Recently, 3D printing is also exploring the possibility of manufacturing high-frequency devices, thanks to the appearance of new materials and the countless alternatives that can be achieved in the design of any circuit, focusing on 3D structures, such as antennas, waveguide devices and microwave sensors [9,10,11,12,13,14,15,16].

Another possibility that 3D printing can offer in the manufacture of high-frequency devices is the design of the geometry and dielectric permittivity of planar substrates for many applications, avoiding limiting constraints of pre-established characteristics of commercial substrates determined by each manufacturer [17,18]. Previous studies have been carried out on the use of 3D printing to create high-frequency substrates using fused deposition modeling (FDM) technology [19,20]. In these works, FDM advantages are exploited like its low cost, the ease of using several materials with different properties and the possibility of modifying printing parameters, such as material filling density [19] or layer height to create multilayer structures [21]. The main drawbacks of this technology are the lack of surface precision of the printed parts and the generation of internal air zones or bubbles in the superposition of the layers, creating heterogeneous sheets [19,22,23]. These are due to the fact that the FDM manufacturing process builds the 3D part by joining the filament threads layer by layer, having as a physical manufacturing limit the diameter of the nozzle that extrudes the thermoplastic material. In this actual work, stereolithography (SLA) technology is used to improve the precision and surface quality of 3D printed objects by using a polymeric resin, which can be of great interest in the design of high-frequency planar circuits. These SLA techniques have been successfully used in the implementation of antennas for 5G and C-band applications as in [24,25,26]. Since all the commercial resins available for this type of technology are not specifically intended to implement microwave networks, first it is necessary to determine their electrical characteristics (relative dielectric permittivity constant and loss tangent). In addition, since no metallic resins are commercially available for SLA, it is necessary to rely on metallization techniques for 3D printing parts in order to implement microwave circuits. The metallization techniques used consist of adhering copper sheets [19] to the substrate, using a metal spray coating [27,28] or through an electroplating process [29,30,31], as implemented with conventional commercial high-frequency substrates [32], in complex waveguides [33] or in additive manufacturing parts [34].

In addition, it is necessary to guarantee that the whole implementation process of the piece results in a consistent and reliable structure. The multistep manufacturing process of the circuit comprises, on the one hand, the fabrication of a substrate based on a structure of polymeric resins joined in successive layers by an ultraviolet (UV) light source, and on the other, the use of different metallization techniques either with epoxy adhesives to join the copper plates or through an electroplating process. Errors in any of the manufacturing stages would compromise the structural integrity of the resulting radiofrequency circuit. The most common ones would be voids or bubbles between the layers of the cured polymer, at the substrate–adhesive–copper or substrate–copper interfaces in the areas of electroplating metallization, excess adhesive or inhomogeneity in density of the material layers. For this reason, a structural analysis is carried out using non-destructive ultrasonic techniques, since they are fast and cheap, and can be used without damaging the implemented devices. Circuits are evaluated using time and frequency domain analysis techniques based on deconvolution and resonance spectroscopy [19,35,36,37,38,39,40].

The main objective of this work is to show how using 3D printing techniques, and in particular SLA techniques, the designer of high frequency planar circuits can incorporate the characteristics of the substrate to be used in each region of the circuit in the design process of the device, without the limitations entailed by the need to choose from a limited range of commercial substrates with certain physical and electrical properties. For this, the low-cost technological process followed for the manufacture of substrates with resin and SLA techniques is shown first, as well as two alternatives for the metallization of the transmission lines on them. Next, the electrical characterization of the resin and the considered metallization processes is carried out, obtaining as a result the parameters to be incorporated into the simulation tools used for the design of high frequency circuits. This work shows the possibility of using low-cost SLA 3D printing technology in the design of high-frequency substrates with superior quality in precision and surface finish of the 3D part compared to FDM technology. To validate this technology, a proof of concept complex implementation of a simple microwave low pass filter device is fabricated and measured, and its structural analysis is carried out using ultrasonic techniques. Following the manufacturing and metallization processes employed, it would be possible to implement much more elaborated structures for the design of high-frequency circuits, such as waveguide filters through the use of periodic structures [33,41], where a higher resolution in the 3D pieces would allow for increasing the operating frequency.

This work is organized in four sections. Section 2 describes the resin used in this study, along with the characteristics of the low-cost 3D printer and the manufacturing and metallization processes of a high-frequency substrate. It also depicts the system used to perform the ultrasonic structural analysis and continues with the electrical characterization of the substrate. Section 3 includes the design, implementation and characterization of a stepped impedance low pass filter, using a novel multi-height substrate to enhance the features of the device. Finally, the conclusions of this work are outlined in Section 4.

## 2. Materials and Methods

### 2.1. Resin

In this work, a standard commercial 3D JAKE resin in dark blue color has been characterized electrically and mechanically for use in the manufacture of radiofrequency circuits. This type of resin is photosensitive at 405 nm and is composed of a mixture of acrylates (96.95%), photoinitiators (3%) and a pigment (0.05%) to color the material. The recommended printing parameters of this resin determined by the manufacturer are the following: a layer height of 0.05–0.1 mm, an exposure time per layer of 8 to 14 s and 5 to 10 lower layers with an exposure time of 45–60 s, to make a good adhesion to the 3D printing surface [42].

### 2.2. 3D Printer

**Figure 1:**
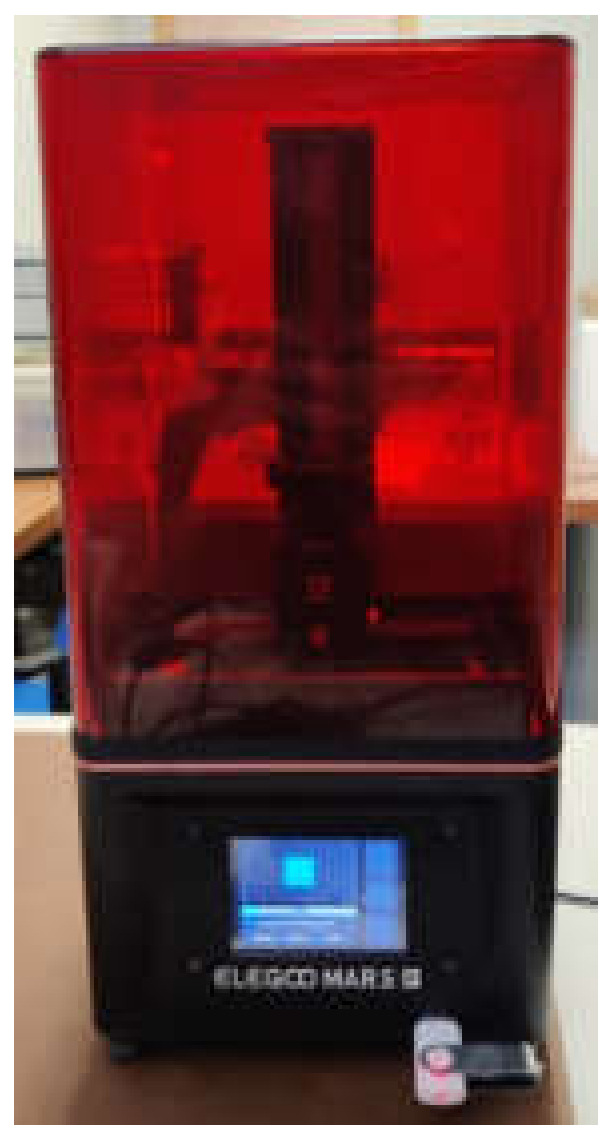
Photograph of the printer employed.

The 3D printer used to manufacture the high-frequency devices with the resin presented in the previous section is the Mars PRO 3D (from Elegoo), as shown in Figure 1. This type of low-cost 3D printer (around €200), allows for printing a maximum volume of 120 × 68 × 155 mm^3^ with a resolution of 47 µm on the horizontal axis and 10 µm on the vertical axis [43].

This 3D printer employs MSLA (Mask Stereolithography) technology that uses an LCD (Liquid Crystal Display) screen as a mask for ultraviolet light, which allows for printing a wide variety of resins with different properties and handling different printing options which affect the final quality of the 3D printed objects including: layer height, exposure time per layer, number of lower layers, and exposure time of lower layers. To adjust the different parameters of the resin used in the printer and model the 3D object by layers, the three-dimensional software Chitubox from CBD-Tech [44] was used, as shown in Table 1.

### 2.3. Manufacturing Process

The manufacturing process is carried out with the resin and the 3D printer that have been described in the previous sections. Once the piece has been manufactured, it is immersed in isopropyl alcohol (IPA) for approximately 20 min. The IPA bath is used to remove excess resin that was not cured during the 3D printing process and to smooth out any roughness in the structure. Finally, the piece is finished solidifying with UV rays for 10 min, giving greater consistency and strength to the design. The fabricated structures are then metallized following a copper foil bonding process for flat surfaces and an electroplating method for irregular surfaces.

#### 2.3.1. Substrate Metallization Using a Glued Copper Layer

**Figure 2:**
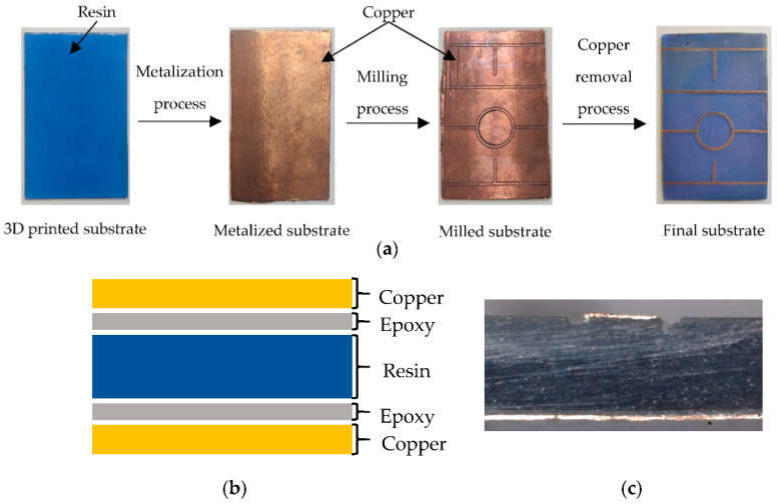
Printed circuit board manufacturing process with glued copper layer: (**a**) step by step description; (**b**) cross-section of the manufactured substrate; (**c**) transmission line end cross-section.

This metallization process consists of gluing two 35 μm copper sheets from Basic Copper on both sides of the substrate, using a thin layer of non-conductive epoxy glue 2216 B/A GRAY from 3M applied manually with a brush. For a correct union, a pressure of 15.3 kgf/cm^2^ is applied by means of a hydraulic press to the two copper sheets for one hour, with an ambient temperature of 23 °C and a humidity of 30%. Once the epoxy glue solidifies, the substrate is ready to be used in the fabrication of any microwave circuit using a numerically controlled milling machine; in particular, the LPKF Protomat S42 has been used. Figure 2a describes the manufacturing process of the printed circuit and Figure 2b,c show the cross section of the substrate structure after metallization.

#### 2.3.2. Substrate Metallization Using Copper Spray Paint and Electroplating

**Figure 3:**
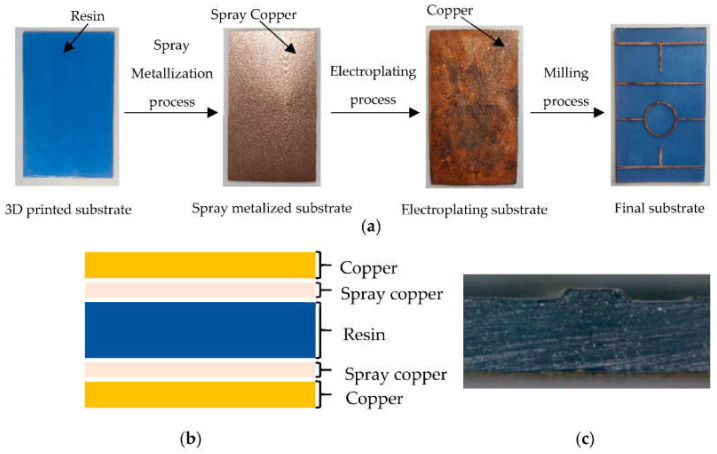
Printed circuit board manufacturing process with copper spray and electroplating: (**a**) step by step description; (**b**) cross-section of the manufactured substrate; (**c**) transmission line end cross-section.

This metallization method consists of creating a first layer of conductive material on the surface of the printed structure, to carry out later the metallization by copper electroplating. The structure is coated first with a layer of Super Shield spray, manufactured by MG Chemicals. This spray contains copper particles that adhere to the substrate surface and form a copper film of approximately 70–100 μm. The curing process for this paint takes approximately 12 h at room temperature and, once it is finished, an electroplating process is carried out to increase the thickness and conductivity of the copper. Thus, the pieces are immersed in an electrically conductive solution, which is composed of copper sulfate, sulfuric acid and some extra additives to soften the copper deposition and increase its brightness [45]. The duration of the metallization process depends on the applied current and the area of the piece immersed in the solution. In this case, a current intensity of 1.5 A/dm^2^ was selected to obtain a copper growth of approximately 100 μm/h. Once the electroplating procedure is finished, the substrate is ready to be used in a numerical control milling machine to manufacture any high-frequency device. Figure 3a describes the manufacturing and metallization process of the printed circuit while Figure 3b,c show the different layers in a cross-section of the implemented substrate.

### 2.4. Structural Analysis

**Figure 4:**
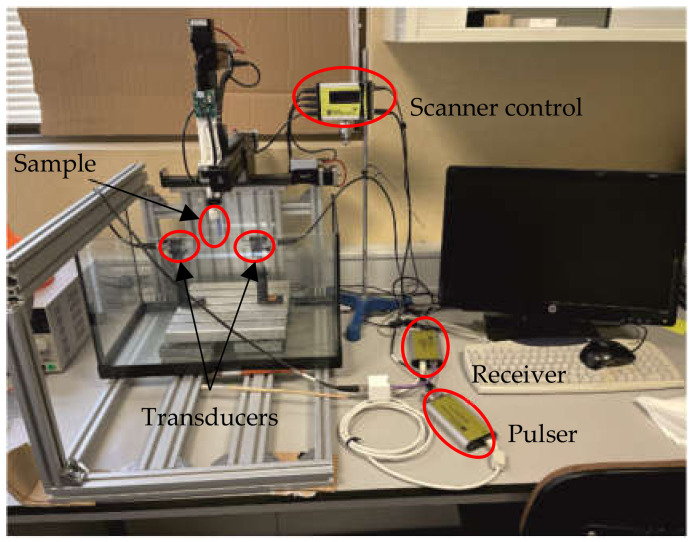
Experimental setup for ultrasonic measurements.

The structural analysis of the different circuits was conducted using immersion pulse-echo ultrasonic non-destructive testing techniques. The circuits were scanned in an immersion basin in distilled water, using a 5 MHz focused transducer V309 from OLYMPUS as pulse-echo transducer. The excitation used was a rectangular 0.2 µs pulse with central frequency of 5 MHz. For each circuit, the XYZ scanner performed a C-scan (2D scans along all the surface), taking A-scans (single measurement at a specific point on the surface of the circuit) every 200 μm. The pulser/receiver used as generator and acquisition equipment was from KTU Electronics [46], with 200 MHz sampling frequency in the pulser to generate the bipolar pulses, and 100 MHz sampling frequency with 10 bits for the acquisition of the samples. Figure 4 shows the described set-up as it is mounted in the laboratory.

The resulting A-scans were processed using two simple time-domain analysis techniques. First, the profile of the front and back surfaces is obtained, which leads to the thickness profile of the circuit. To do so, the time-of-flight (ToF) of the front and back surfaces was obtained using the iterative deconvolution developed in [35], as it allows the estimation of the thickness profile automatically.

The next step of the study was the analysis of the magnitude of the envelope of the processed A-scans at different depths [19]. This can be seen as a sliced picture of the circuit and creates 2D profiles of its inner structure. This process reveals discontinuities and provides a preliminary estimation of the homogeneity of the structure. Significant differences in magnitude and new echoes appear where the acoustic impedance changes, which happens specially with voids, bubbles and interfaces between materials, that is, at interfaces between resin/adhesive/copper, resin/water/copper (delaminations in the borders), copper/water or resin/water (inclusions on the surfaces) or resin/air (inner bubbles embedded during the printing process).

### 2.5. Electrical Characterization at Microwave Frequencies

To proceed with the characterization of the resin electrical properties, several microwave networks with different frequency responses and physical dimensions were manufactured. The scattering parameters of these high-frequency calibrating elements were measured up to 8.0 GHz using an Anritsu 3680 universal test fixture. They were used to extract through an optimization process, using Advance Design System (ADS) by Keysight simulating tool, the value of the main electrical characteristics for the substrate and fabrication procedure which are: the relative dielectric constant of the resin, the loss tangent of the material, and the conductivity associated with the metallization method.

**Figure 5:**
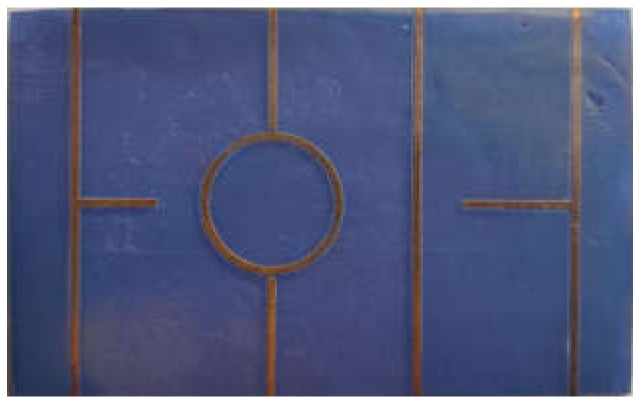
Photograph (50 × 80 mm^2^) of the calibrating elements employed to measure and extract the electrical parameters of the resin substrate material and the fabrication process. Transmission lines are 850 μm wide and 50 mm long, and gaps in the ring resonator network are 360 μm.

As shown in Figure 5, the set of elements is formed by a transmission line and several resonant networks of different physical dimensions and characteristics: two quarters of a wavelength open-ended stubs with different lengths and a ring resonator weakly coupled to the input and output ports. The physical dimensions of the calibrating networks are summarized in Table 2 (they were determined making use of a Dino-lite digital microscope). These exact same elements were employed on all manufactured substrates. Substrates with different heights (*h* = 1.0 mm and *h* = 1.6 mm) were analyzed to provide an accurate characterization of the electrical parameters.

#### 2.5.1. Glued Copper Layer Metallization

**Figure 6:**
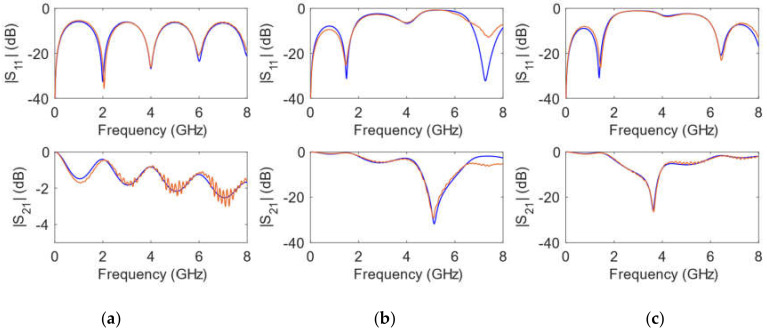
Simulated (**blue**) and measured (**orange**) calibrating elements magnitude of scattering parameters for a *h* = 1 mm substrate height. (**a**) transmission line; (**b**) short stub; (**c**) long stub.

**Figure 7:**
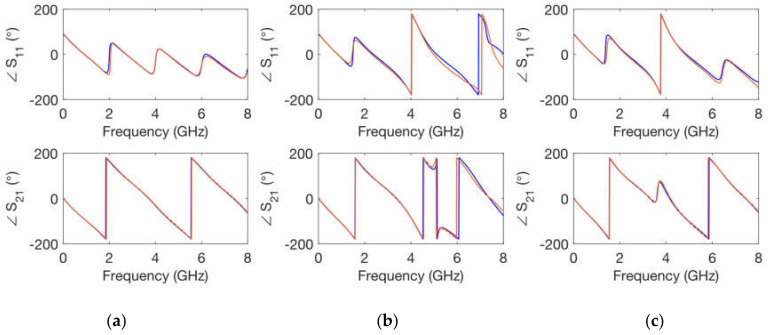
Simulated (**blue**) and measured (**orange**) calibrating elements phase of scattering parameters for a *h* = 1 mm substrate height. (**a**) transmission line; (**b**) short stub; (**c**) long stub.

**Figure 8:**
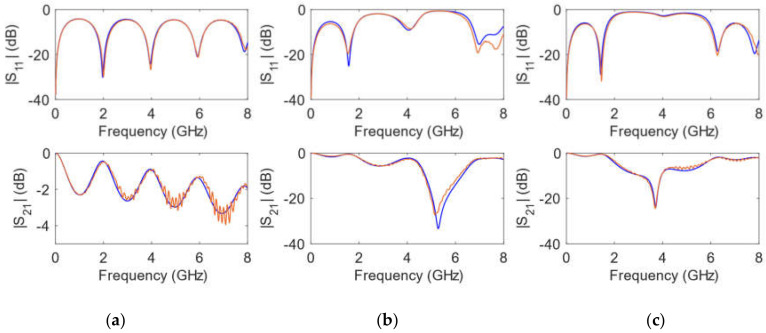
Simulated (**blue**) and measured (**orange**) calibrating elements magnitude of scattering parameters for a *h* = 1.6 mm substrate height. (**a**) transmission line; (**b**) short stub; (**c**) long stub.

**Figure 9:**
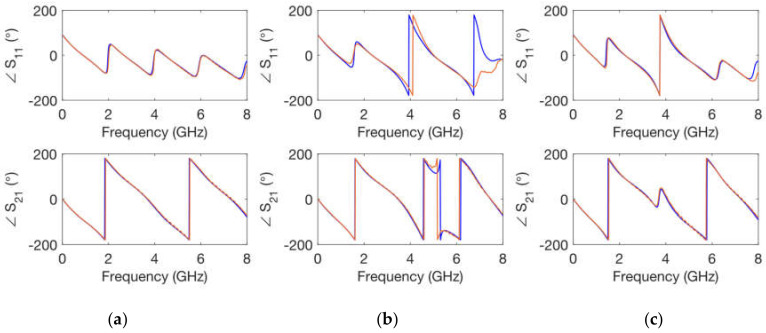
Simulated (**blue**) and measured (**orange**) calibrating elements phase of scattering parameters for a *h* = 1.6 mm substrate height. (**a**) transmission line; (**b**) short stub; (**c**) long stub.

Once the substrates were printed, a first set of calibrating elements was implemented by means of the process described in Section 2.3.1. Figure 6, Figure 7, Figure 8 and Figure 9 show the comparison between measurements and simulations of the calibrating networks given in Figure 5 (transmission line and both open-ended stub networks).

The simulations were carried out in ADS by means of circuit elements with the exact physical dimensions yielded by the fabrication process. In addition, the conductivity of copper σ = 5.8 × 10^7^ S/m was considered in the transmission line elements. The values for the relative dielectric permittivity constant and for the loss tangent parameter of the substrate were optimized. Thus, an exact replica of the measured scattering parameters was obtained, both in magnitude and in phase for the whole frequency range considered. As can be seen in Figure 6, Figure 7, Figure 8 and Figure 9, optimized simulations and measurements, both in phase and in magnitude, are in complete agreement up to 8.0 GHz. The optimized responses were achieved by considering a relative dielectric permittivity constant of ε_r_ = 3.25 and a loss tangent factor of tanδ = 0.03. Some remarks that can be pointed out from Figure 6, Figure 7, Figure 8 and Figure 9 are that the transmission line width employed *w* = 0.85 mm does not correspond to a 50 Ω characteristic impedance in any of the substrates (as it is evident from the |S_11_| parameter), and that the resonance produced by the open-ended short stub appears around 5.2 GHz, while that created by the open-ended long stub occurs at 3.7 GHz. Phase simulations and measurements are shown to prove that electrical lengths (dependent on the physical dimensions and the relative dielectric permittivity constant) in all calibrating elements match.

**Figure 10:**
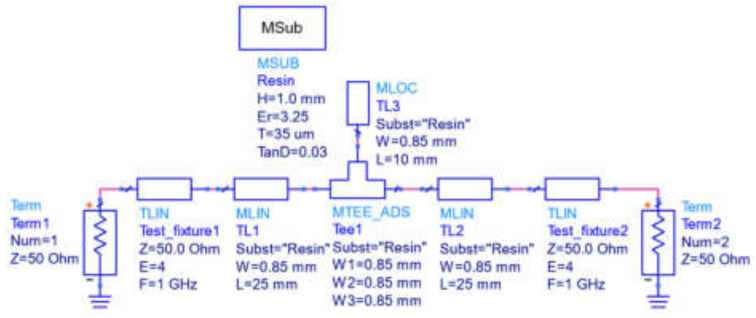
Circuit elements employed to simulate the response of the 10 mm long quarter wavelength open-ended stub calibrating network.

Finally, Figure 10 shows as an example the circuit elements employed to simulate the response of the network with the longest quarter wavelength open-ended parallel stub. It is worth noting that the input and output ports include extra lossless 50 Ω characteristic impedance transmission line sections to account for the additional electrical lengths introduced by the Anritsu test fixture connections not included in the vector network analyzer calibration procedure. In addition, as justified in Section 2.3, the substrate is assumed homogenous despite the introduction of the 30 μm thick epoxy layer to attach the copper metallic top and bottom sheets (the conductivity is set to that of copper σ = 5.8 × 10^7^ S/m to perform all simulations).

**Figure 11:**
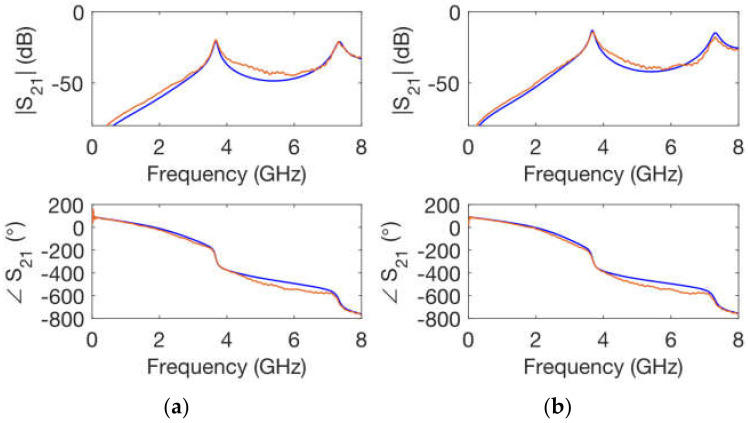
Simulated (**blue**) and measured (**orange**) ring resonator network magnitude and phase of the transmission coefficient: (**a**) *h* = 1.0 mm substrate height; (**b**) *h* = 1.6 mm substrate height.

The scattering parameter simulations of the ring resonator networks for both substrate thicknesses were carried out (assuming ε_r_ = 3.25 and tanδ = 0.03) to verify the correct extraction of the electrical characteristics. Figure 11 shows that simulations and measurements are in agreement. Simulations do not only match accurately the resonant frequencies of the calibrating ring networks, but also agree in the determination of the measured *Q* factor of all resonances.

#### 2.5.2. Resin Substrate Metallization Using Electroplating

One of the main disadvantages of the metallization process, characterized in the previous subsection, is that the attachment of the metallic copper layer using epoxy, cannot be achieved with satisfactory results if the structure of the circuit is more complex than the one shown in Figure 5 (as it will be highlighted in Section 3). As an attempt to avoid this circumstance, calibrating elements of Figure 5 were manufactured employing the metallization methodology described in Section 2.3.2.

**Figure 12:**
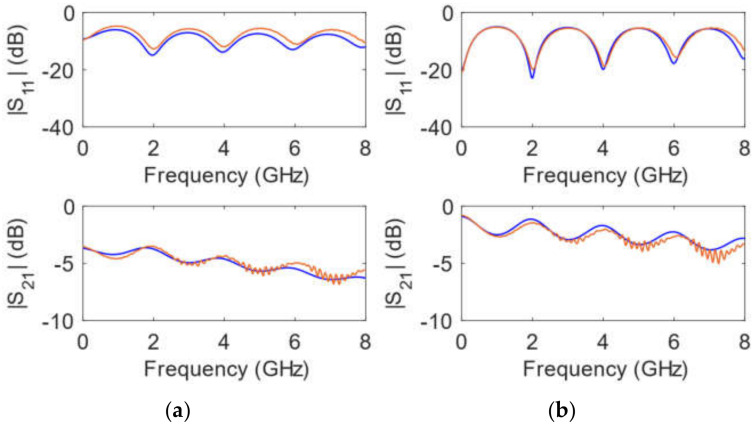
Simulated (**blue**) and measured (**orange**) magnitude of the scattering parameters for a 50 mm long transmission line (*w* = 0.85 mm) for the *h* = 1.6 mm substrate height: (**a**) just metallic paint is applied; (**b**) after electroplating is also carried out.

Figure 12 shows the comparison between simulations and measurements of the calibrating 50 mm long transmission line, which was originally implemented using just the metallic paint and then further metallized with an electroplating process (as described in Section 2.3.2). Simulations were carried out by optimizing the value of the metal conductivity. This process yielded an estimated conductivity of σ = 4.0 e3 S/m for the transmission line fabricated by the deposition of metallic paint and of σ = 8.0 e4 S/m when this process was followed and finished by electroplating. As expected, results in terms of losses are not as appropriate as those shown in Figure 8a. At a particular frequency of 6.0 GHz, losses obtained from the transmission line of Figure 8a are 1.2 dB, which can be assumed produced by the dielectric resin material. Again at 6.0 GHz, from Figure 12, losses are before and after electroplating process 5.3 dB and 2.5 dB, respectively. Considering that, at 6.0 GHz, 1.2 dB correspond to losses in the resin material, the losses attributed to the metallic paint are 4.1 dB, and those coming from the conductors after electroplating 1.3 dB.

## 3. Results

### 3.1. Filter Design

To demonstrate the versatility and strengths of stereolithography 3D-printing techniques for enhancing the characteristics of microwave planar circuits, a low pass filter prototype is fabricated. The filter is designed by considering a traditional stepped-impedance approach [47], where series inductances are synthesized with high characteristic impedance short transmission line sections, and parallel capacitances are obtained by including low characteristic impedance short transmission line sections.

**Figure 13:**
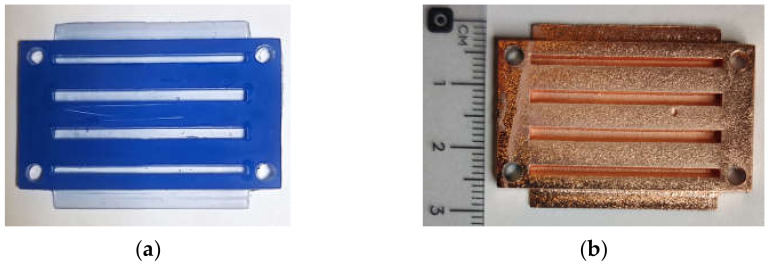
Fabricated low pass filter prototype: (**a**) before the deposition of copper metallic paint and electroplating process on the ground plane; (**b**) after the metallization procedure.

In [19], the performance of the proposed filter design was improved by increasing the highest characteristic impedance available in the manufacturing process. This was accomplished by designing a substrate with lower density filling pattern printed areas under the short transmission line sections used to implement the series inductances. In this work, we propose the use of stereolithography printing for the fabrication of a substrate with varying heights depending on the circuit element to be synthesized. Thus, for manufacturing high impedance sections, 3.0 mm high substrate areas are employed, while, under the low impedance sections, the substrate is fabricated with a height of 0.4 mm. In this way, assuming that restrictions in the milling process are *w* = 0.5 mm for the minimum achievable line width and *w* = 10 mm for the maximum advisable line width, it is possible to determine the range of available values for the transmission line characteristic impedances to be fabricated on the substrate. Table 3 summarizes these calculations and compares them to those for a constant *h* = 0.70 mm high substrate. A 9 element 0.1 dB equal ripple Chebyshev low pass filter was designed, simulated and manufactured following the popular stepped impedance configuration, where alternating high and low characteristic impedance transmission line short sections are employed to synthesize the inductors and capacitors of the lumped element filter prototype. Figure 13 shows the bottom view (ground plane) of the substrate printed using the resin characterized in Section 2. The height of each section in the substrate is paired with its corresponding short section transmission line width on the top size. The structure includes two flap-like 3.0 mm long sections in order to implement 50 Ω input/output ports in the network and accommodate the design conveniently for its characterization using the Anritsu 3680 universal test fixture. Figure 13 also shows the printed substrate metallized as described in Section 2.3.2, and the inclusion of fiducial markers to permit the correct positioning of the structure during the milling process of the transmission line sections on the top side of the filter.

**Figure 14:**
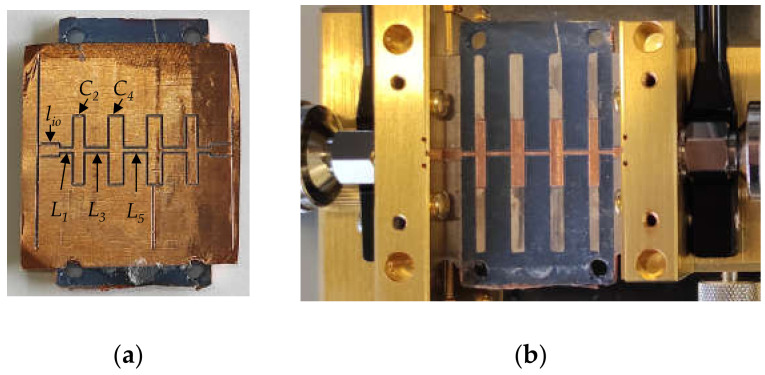
Final implementation of the low pass filter prototype: (**a**) after milling process of the top low and high impedance sections; (**b**) filter characterization using Anritsu 3680 universal test fixture.

Figure 14 shows on one side the final implementation of the filter after the milling process of the top layer transmission line short sections, and before the excess of copper sheet is removed, and, on the other side, the measurement setup of the filter embedded in the test fixture. Filter element dimensions are given in Table 4 (the filter is symmetrical).

### 3.2. Filter Structural Analysis

**Figure 15:**
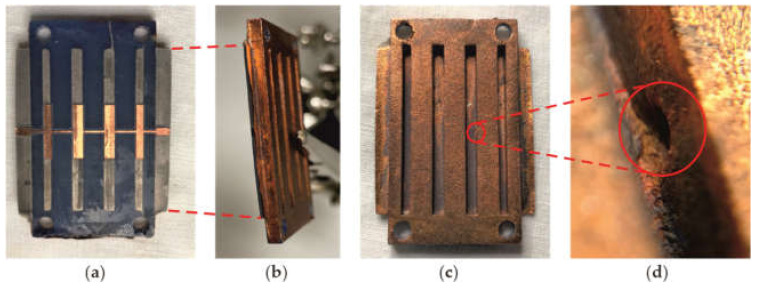
Circuit under analysis: (**a**) top surface; (**b**) details of delamination in the boundary; (**c**) bottom surface; (**d**) details of inclusion/void.

The circuit under ultrasonic analysis is shown in Figure 15, with some points and details of particular interest that will be later discussed.

**Figure 16:**
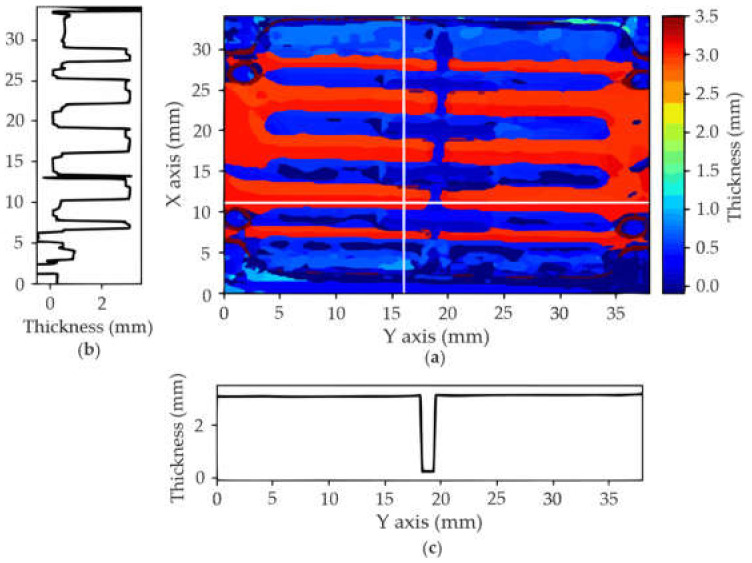
C-scan: (**a**) thickness profile; (**b**) X section; (**c**) Y section.

Figure 16 shows the thickness profile for the whole surface (Figure 16a), including profiles along particular scanning lines on the X (Figure 16b) and Y (Figure 16c) axes, marked with white lines in Figure 16a. Note that ToF are converted to distances using the speed of sound in the resin, obtained in a different experiment, and not taking into account the copper layers due to their very short thickness compared to that of the resin layer (therefore, results are only approximations).

**Figure 17:**
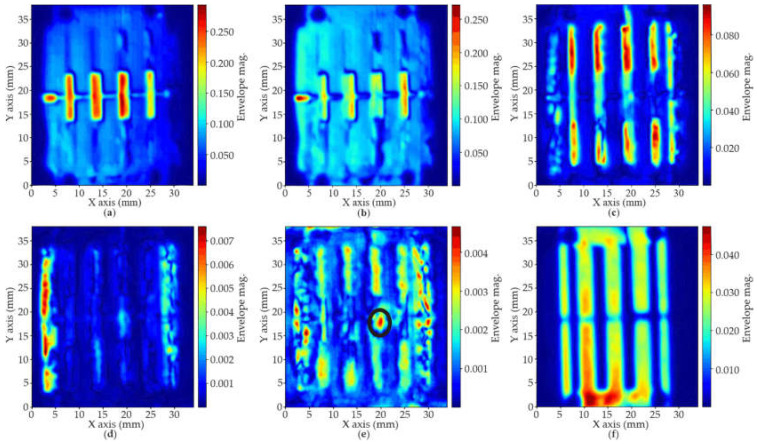
Examples of 2D profiles of the inner composition at different depths. Inner scans provide useful information regarding the homogeneity of the fabricated structure and reveal the presence of voids, bubbles and other irregularities.

Finally, Figure 17a–c show that there are no significant errors on the top surfaces, neither on the copper layer (drilled or not) nor on the resin layer. In addition, Figure 17d shows that there are no defects in the thin resin layer (between 0 and 0.4 mm). However, some unexpected reflections appear at the most lateral layers. This effect is much more evident in Figure 17d,e. It is due to the delamination suffered between the bottom copper layer and the resin, probably due to the cutting process that damaged that border, and also to the manipulation of the sample during the scanning set-up, which resulted in the unsticking of the copper at some points (see Figure 15b).

Figure 17e also reveals a void (marked with a black circle, around 19 mm in the X axis and 17 mm in the Y axis) on the bottom surface between the thin and thick layers (see Figure 15c,d). Notice that, although appearing on the slice around 1.2 mm depth, these defects correspond to approximately 6 mm in depth, but, because the gaps are filled with water, what can be seen are the resonances that appear at double the depth (note also that the magnitude of the reflections at that depth is significantly low). Finally, Figure 17f reveals that the bottom layer is quite regular, indicative of the success of the copper deposition process. No other relevant defect or inclusion has been found inside the material, confirming the homogeneity achieved by the printing process.

### 3.3. Filter Electrical Response

**Figure 18:**
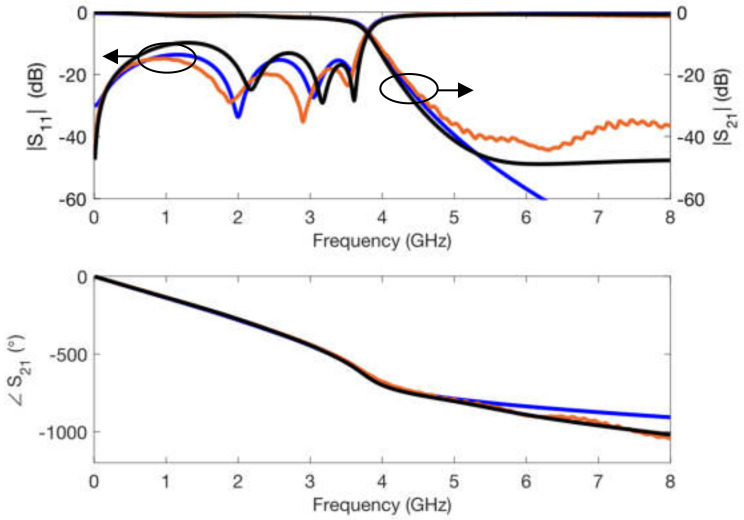
Circuit simulations (**blue**), electromagnetic simulations (**black**) and measurements (**orange**) of the scattering parameters for the low pass filter prototype.

The response of the filter is shown in Figure 18, where a good agreement between circuit and electromagnetic simulations and measurements can be observed. Circuit simulations were carried out using ADS by Keysight, while the electromagnetic response was simulated with HFSS by Ansys. It is worth noting that the instances employed for the circuit simulation response are not conceived for complex geometries like the one presented in Figure 14. For example, discontinuity elements included between two consecutive transmission line sections of different widths implemented with substrates of different heights are just considered for a uniform height substrate, which leads to inaccuracies in the expected results. On the other hand, circuit simulations performed do not take into account possible coupling effects between consecutive parallel capacitance short sections. These coupling effects lead to an |S_21_| transmission parameter floor of around −40 dB in the 6.0 to 8.0 GHz range. Thus, circuit simulations do not reproduce this behavior, while electromagnetic simulations are able to predict it (as is evident from the |S_21_| floor of around −45 dB in the 6.0 to 8.0 GHz range). Similarly, in the |S_21_| transmission parameter phase response, measured and electromagnetic simulations are in excellent agreement, but circuit simulation results are unable to match the phase curve above 5.0 GHz.

Another important fact to point out is that two different metallization techniques have been used to fabricate the final prototype of Figure 14. As discussed in Section 2.5.2, these two techniques yield different conductivity values. The copper layer gluing procedure employed for the top transmission lines is assumed to provide a conductivity close to that of copper, while the copper paint and electroplating process employed in the stepped ground plane gives an estimated conductivity of σ = 8.0 × 10^4^ S/m. Circuit simulators include substrate instances that allow a single conductivity value for both the ground plane and the produced transmission lines. The combined conductivity is estimated to be σ = 3.25 × 10^5^ S/m through an optimization process, and this is the value used in the circuit simulations shown in Figure 18. On the other hand, in electromagnetic simulators, metallic elements with different conductivities can be defined in the network to be analyzed, and so it was done to obtain the electromagnetic simulation results of Figure 18.

**Figure 19:**
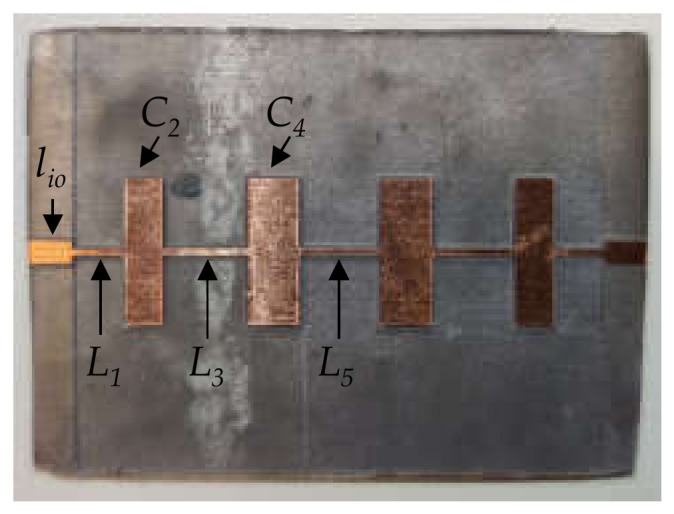
Final implementation of the low pass filter prototype using a resin printed constant *h* = 0.7 mm high substrate.

The same 9 element 0.1 dB equal ripple Chebyshev low pass filter was designed and manufactured using a constant *h* = 0.70 mm high resin substrate (and assuming the same implementation constraints for the milling process). Figure 19 shows a picture of the conventional filter, and Table 5 gives the length of the different elements (the filter is symmetrical). The filter measures 35.62 mm long which is 55.4% larger than the length of the same low pass filter design (22.92 mm) shown in Figure 14.

**Figure 20:**
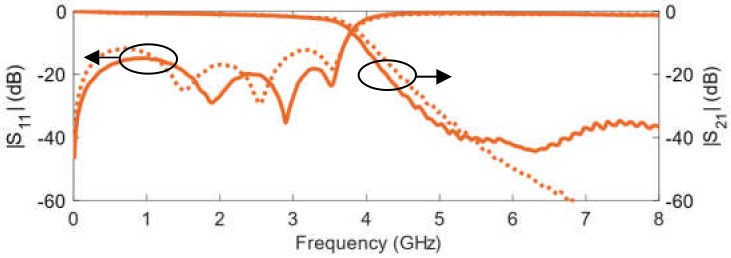
Measured scattering parameters for the stepped substrate low pass filter (**solid line**) and the conventional design on a constant 0.7 mm high substrate (**dotted line**).

The comparison between the measured scattering parameters for both filters is given in Figure 20. Results are very similar in terms of filter performance. The traditional design does not exhibit the |S_21_| floor at around −40.0 dB, although, as stated above, it is 55.4% longer than the stepped substrate implementation.

Table 6 summarizes the comparison between the stepped substrate low pass filter and the conventional design. The electrical performance of both filters is very similar, with the traditional design showing a better response in the rejection band, at the expense of being 55.4% larger than the filter on the proposed stepped substrate.

## 4. Conclusions

This research shows the use of 3D-printing techniques employing SLA for the manufacture of high-frequency devices, where the printed material acts as a medium through which electromagnetic fields propagate. The electrical characterization process of the commercial resin provides a relative dielectric permittivity value of 3.25 and a loss tangent of 0.03. There is a total agreement between the measurements and the simulations performed for the different calibration elements used to extract the electrical characteristics of the resin. These values are similar to those of the FR-4 material, widely used for the manufacture of low-cost electronic circuits. The metallization of printed substrates with SLA is realized in two different ways: by gluing a copper sheet, and by spraying a paint with copper particles and a subsequent electroplating process, depending on whether the surface to be metallized is flat or if it presents a more complex profile. The design of a low-pass stepped impedance filter is performed in which, to reduce its dimensions, larger heights of the printed substrate are used by means of SLA to synthesize the inductive series elements and smaller ones to generate the parallel capacitances. The characteristics of this novel filter structure are compared with those of an identical design following the conventional procedure for a resin substrate of constant height. The electrical response performance of both circuits is similar, but the filter with a variable height substrate implemented by SLA is 55.4% shorter than the conventional one. Finally, a structural analysis of the implemented devices is carried out using ultrasonic techniques. This analysis reveals that AM by means of SLA offers a finish and homogeneity in the manufactured circuits that is not achievable by FDM techniques, and shows the adequacy of the printing and metallization procedures followed for the implementation of high-frequency circuits.

This work shows how low-cost SLA techniques can be used to manufacture planar microwave circuits, which can be an economical alternative to the implementation of high-frequency devices using machining or photolithography techniques on commercial substrates. The main advantage of SLA techniques over these traditional and extended methods is that the designer can implement substrates with different geometric characteristics in the same structure, depending on the needs of each part of the circuit. This advantage, which allows the designed devices to present improved responses, is accompanied by the need for a metallization and milling process, which must be considered in order to establish the economic viability of SLA techniques compared to the usual manufacturing procedures.

## Figures and Tables

**Table 1:** Printing parameters of the resin 3D JAKE in the Elegoo Mars Pro.

Parameter	Value
Layer height (mm)	0.1
Bottom layer count	5
Exposure time (s)	10
Bottom exposure time (s)	50
Lifting distance (mm)	5
Bottom lifting distance (mm)	5

**Table 2:** Calibrating network physical dimensions for both substrates of different heights.

Parameter	Length (mm)
Transmission line	50.0
Short stub	10.0
Long stub	14.0
Ring resonator diameter	17.0

**Table 3:** Characteristic impedances achievable for a constant height substrate and for the stepped substrate (in both cases, substrates are printed with the resin with ε_r_ = 3.25).

Impedance	*h* = 0.7 mm Substrate	Stepped Substrate *h_max_* = 3.0 mm *h_min_* = 0.4 mm
Z_max_ (Ω) (*w* = 0.5 mm)	92.0	151.0
Z_min_ (Ω) (*w* = 10.0 mm)	12.0	7.4

**Table 4:** Filter dimensions. High characteristic impedance sections are *w* = 500 μm wide, low impedance sections are *w* = 10.0 mm wide and input/output sections are *w* = 1.6 mm wide.

Physical Dimension	Length (mm)
*L_1_*	2.22
*C_2_*	1.47
*L_3_*	3.87
*C_4_*	2.00
*L_5_*	3.80
*l_io_*	3.00

**Table 5:** Conventional filter dimensions. High characteristic impedance sections are *w* = 500 μm wide, low impedance sections are *w* = 10.0 mm wide and input/output sections are *w* = 1.6 mm wide.

Physical Dimension	Length (mm)
*L_1_*	3.40
*C_2_*	2.60
*L_3_*	5.54
*C_4_*	3.58
*L_5_*	5.38
*l_io_*	3.00

**Table 6:** Comparison between the stepped substrate low pass filter and the conventional design.

Parameter	Stepped Substrate	Traditional Design
Total length (mm)	22.92	35.62
Cut-off frequency (*f_c_*) (GHz)	3.59	3.63
Pass band return losses (dB)	>14.9	>12.5
Insertion losses at (2 × *f_c_*) (dB)	37.0	>60.0

## Data Availability

Data available in a publicly accessible repository.
